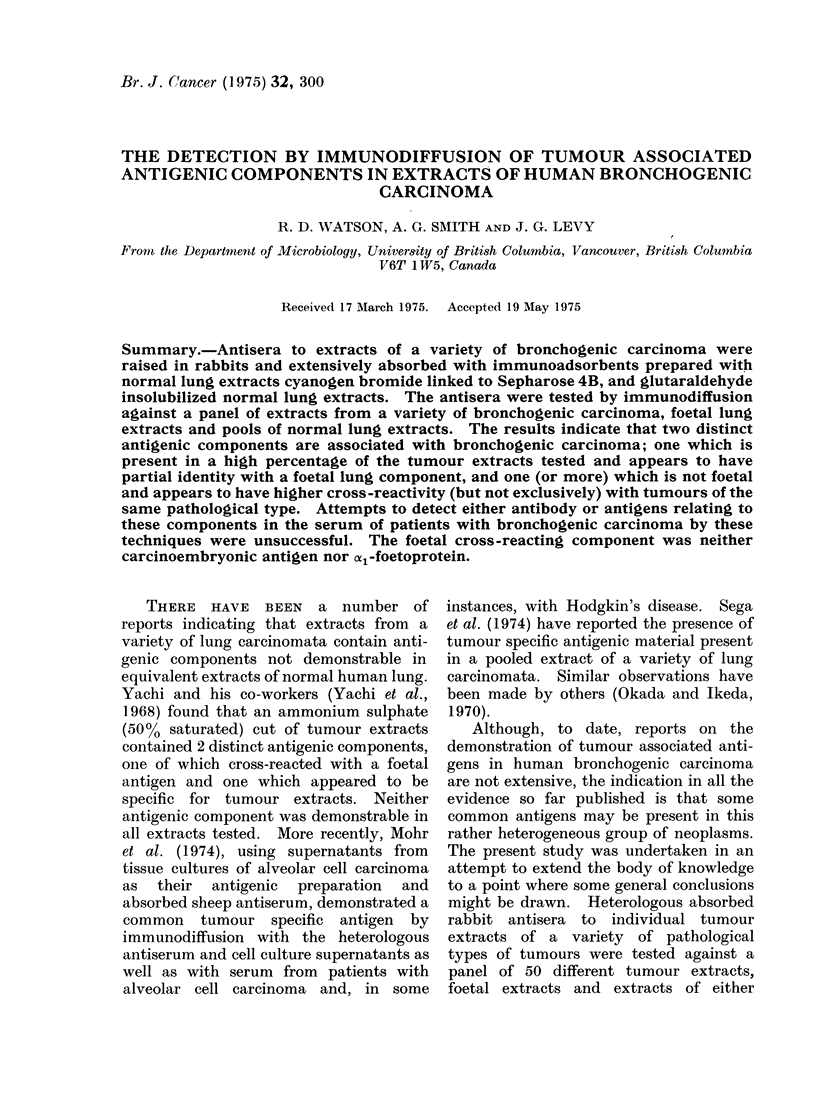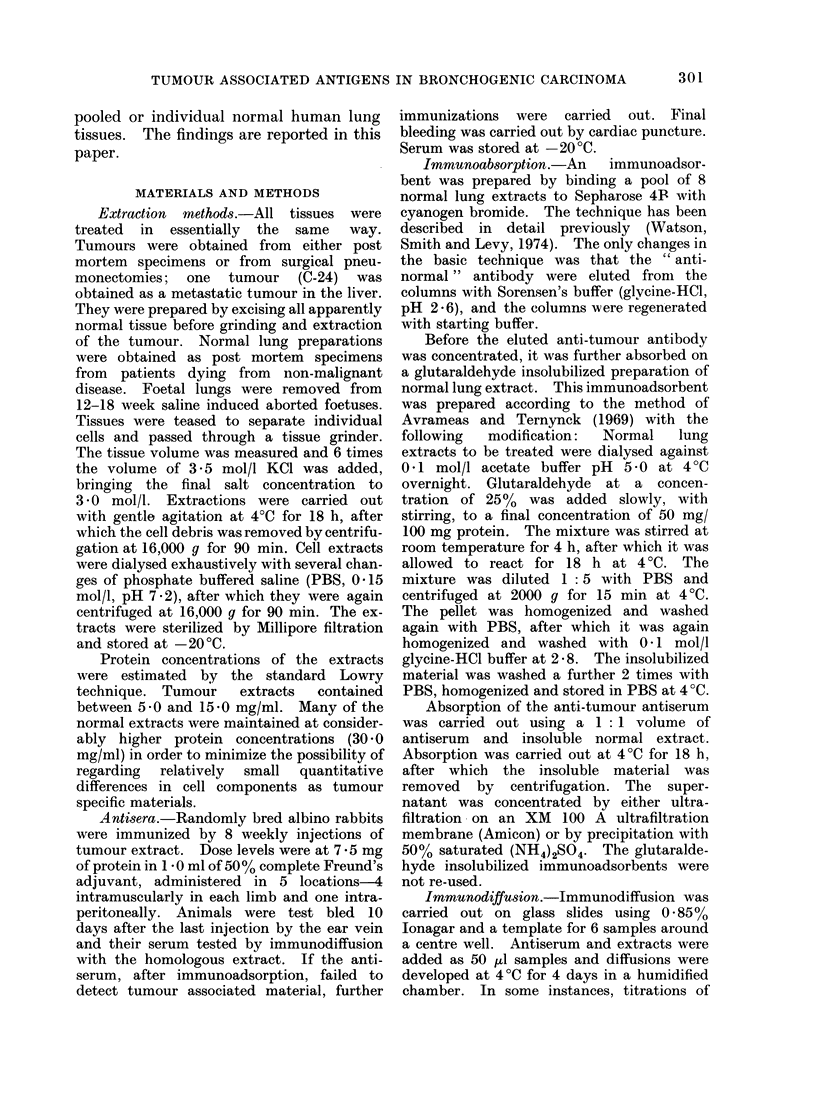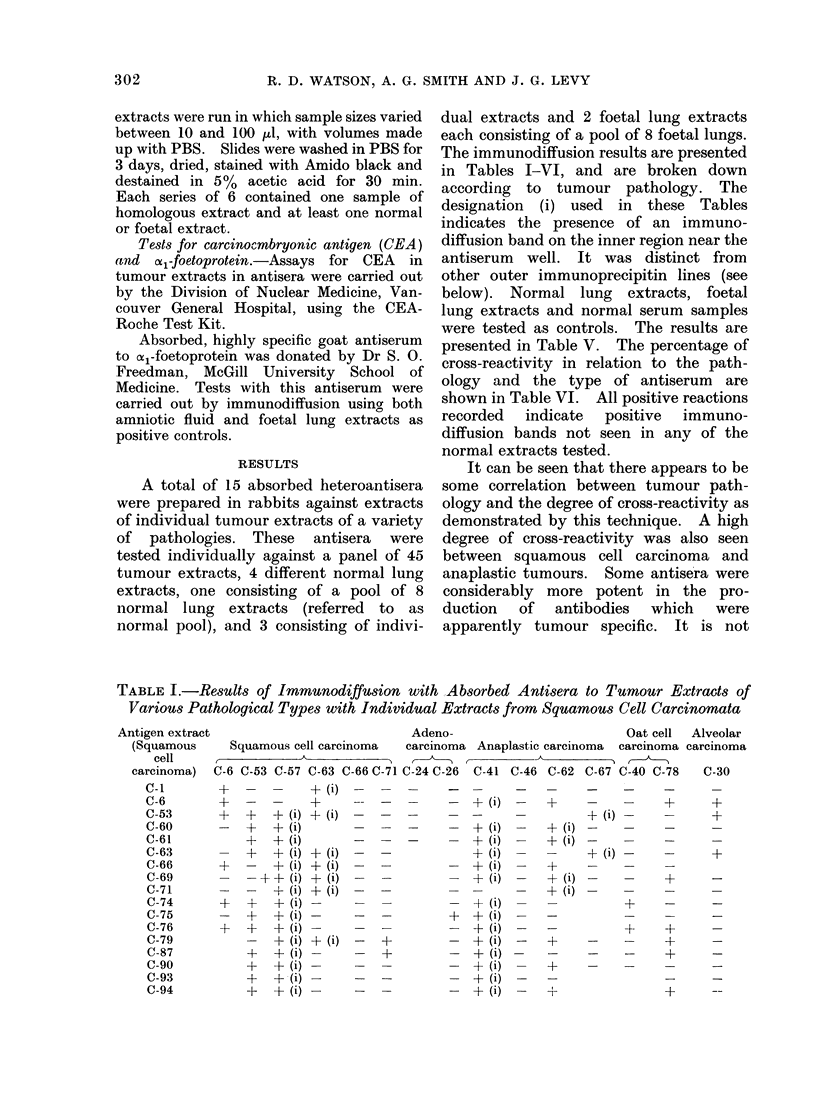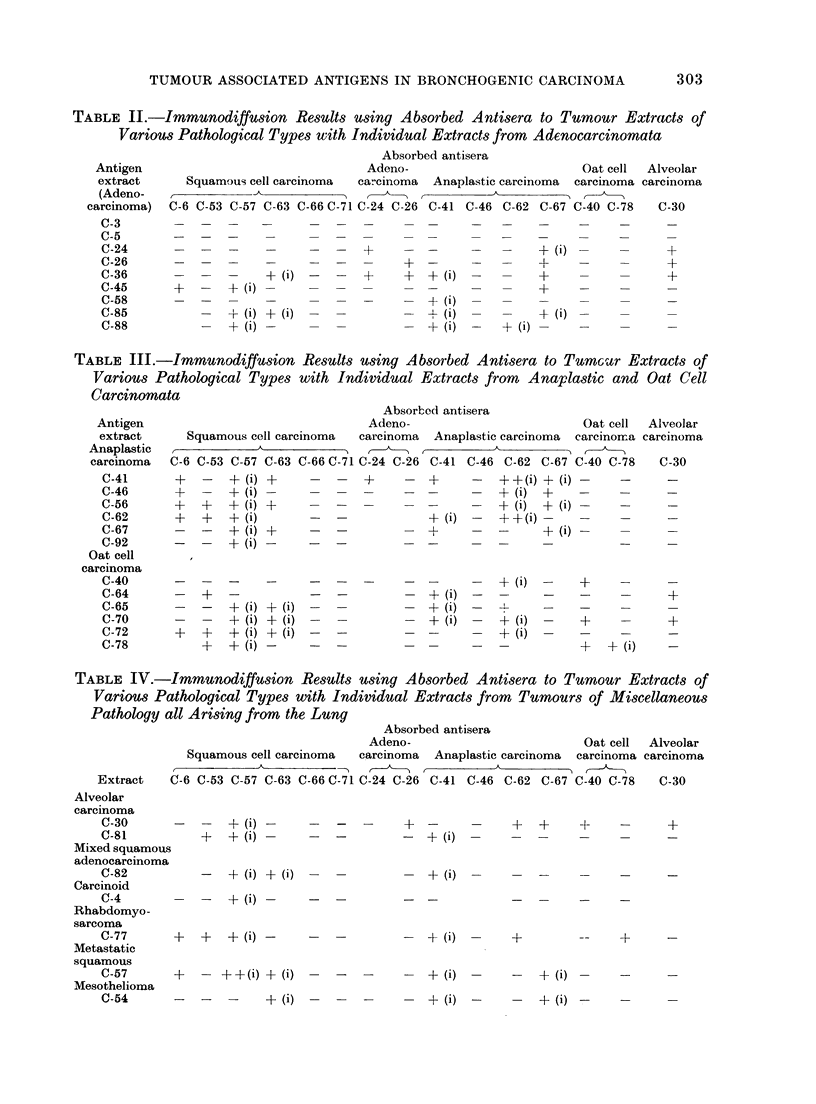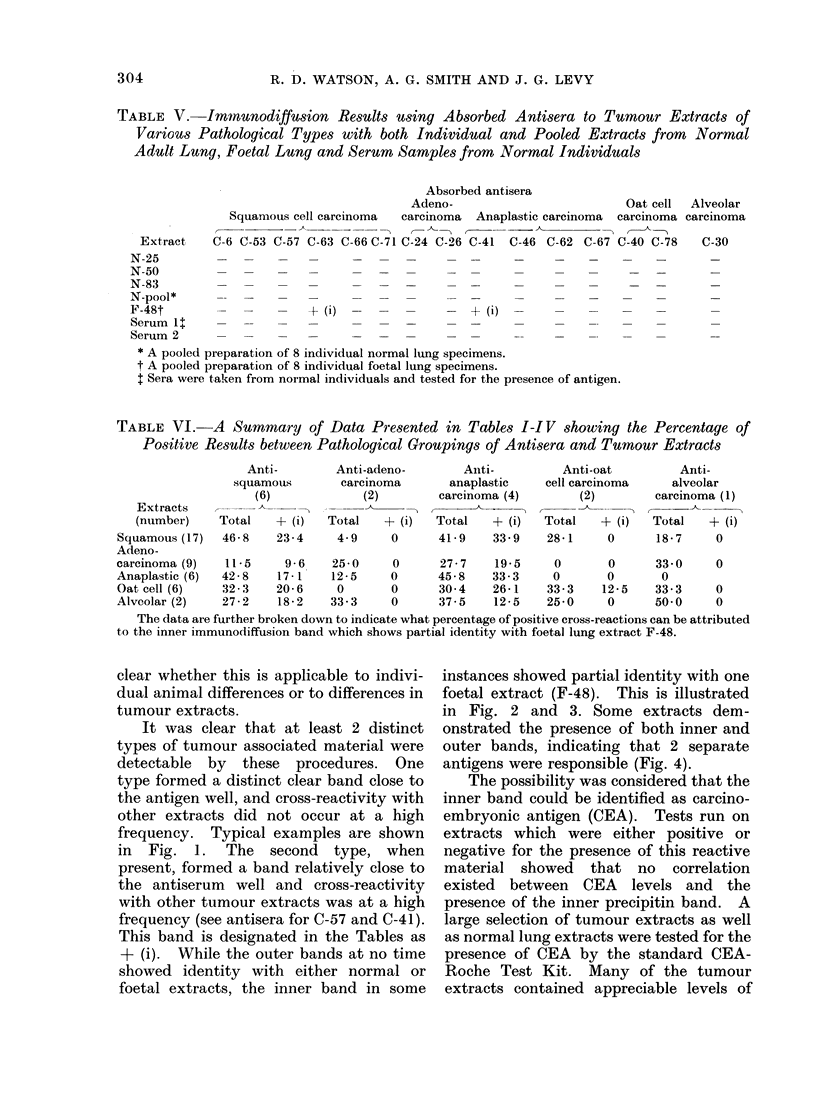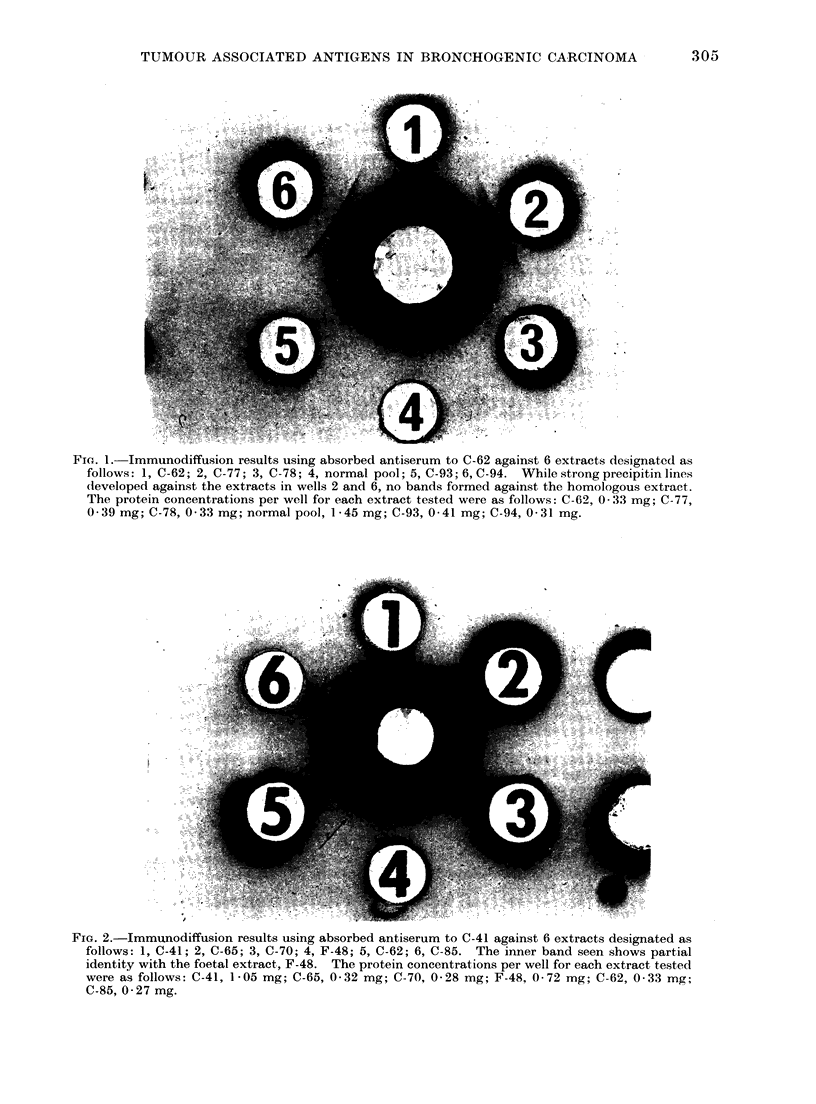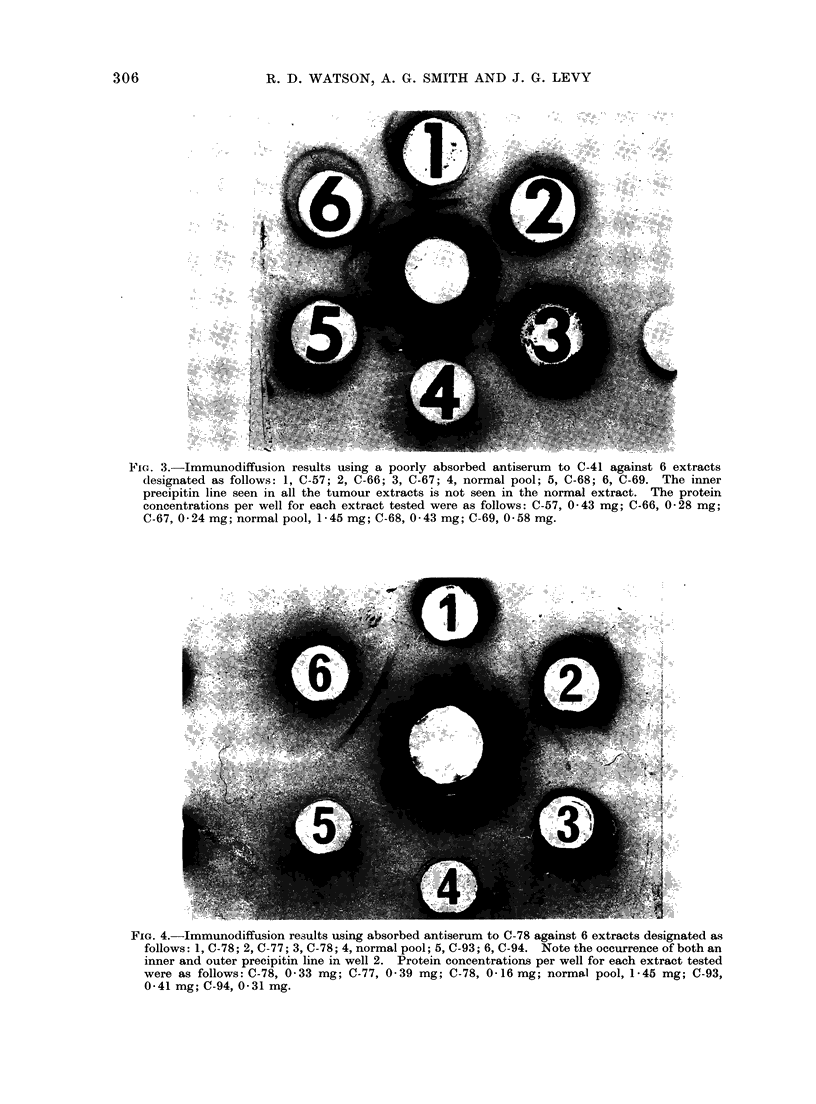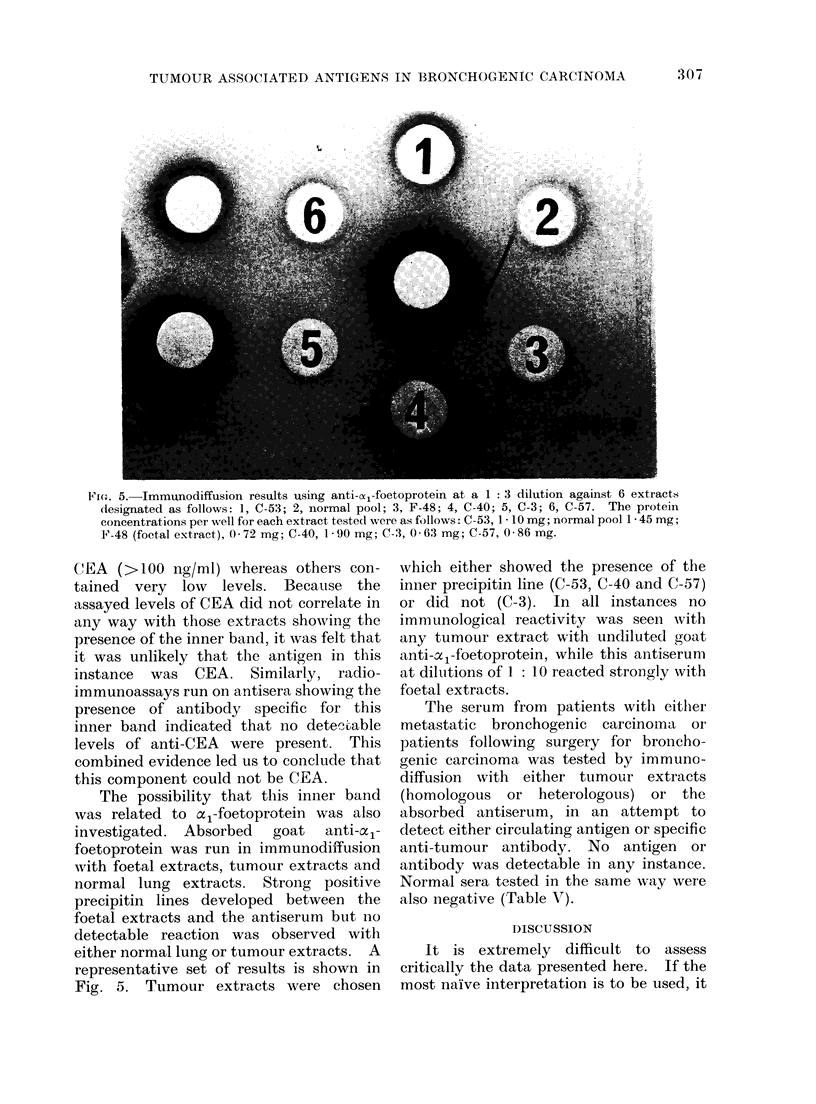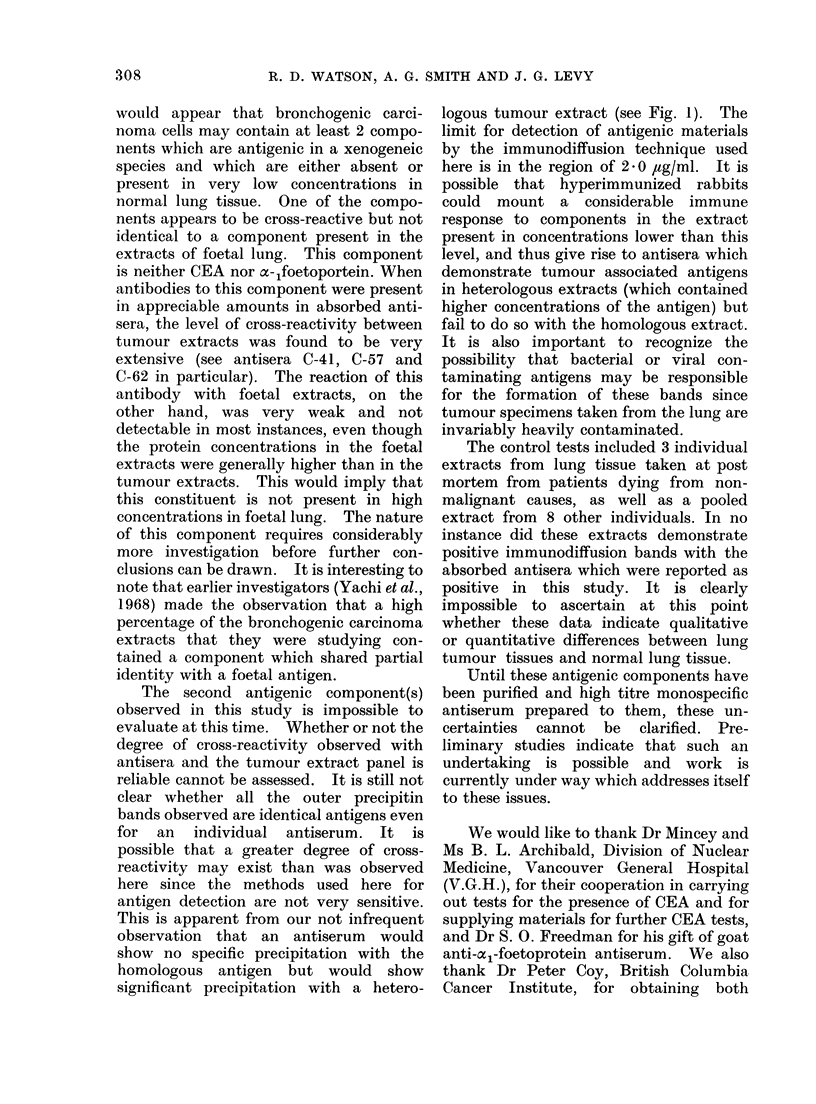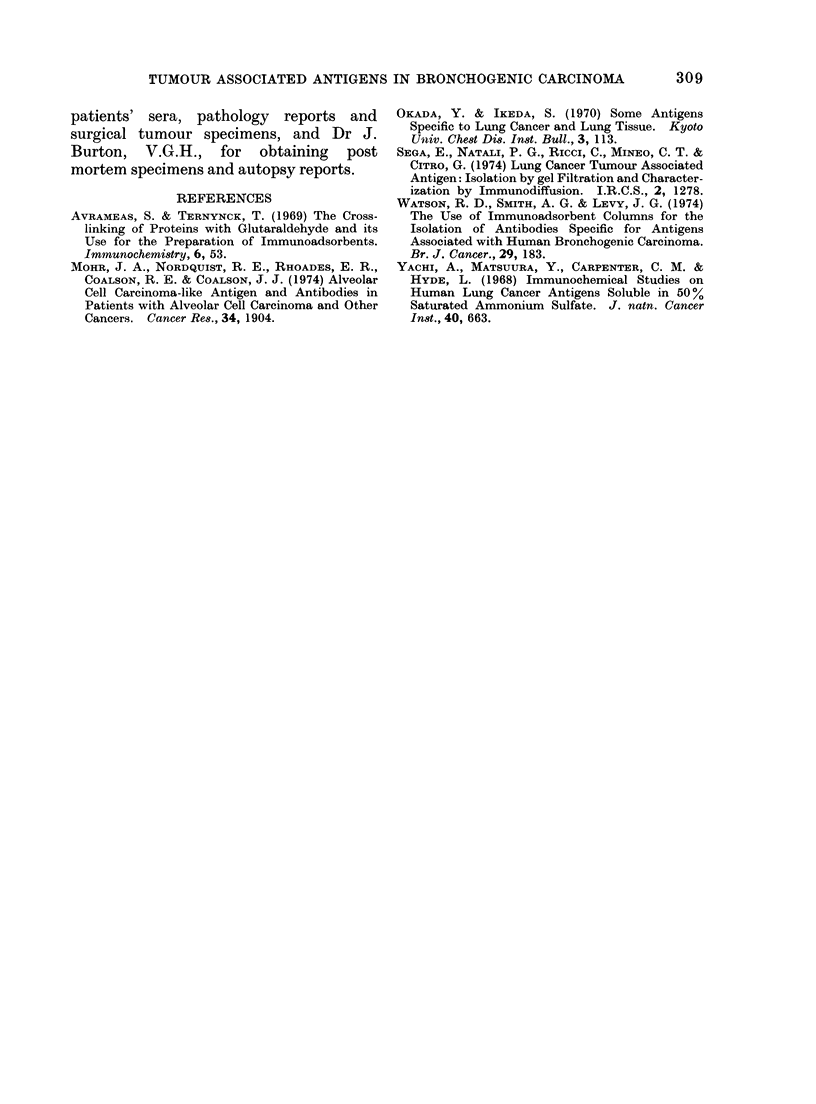# The detection by immunodiffusion of tumour associated antigenic components in extracts of human bronchongenic carcinoma.

**DOI:** 10.1038/bjc.1975.228

**Published:** 1975-09

**Authors:** R. D. Watson, A. G. Smith, J. G. Levy

## Abstract

**Images:**


*Br. J. Cancer* (1975) **32**, 300

### THE DETECTION BY IMMUNODIFFUSION OF TUMOUR ASSOCIATED ANTIGENIC COMPONENTS IN EXTRACTS OF HUMAN BRONCHOGENIC CARCINOMA

R. D. WATSON, A. G. SMITH AND J. G. LEVY

*From the Department of Microbiology, University of British Columbia, Vancouver, British Columbia V6T 1W5, Canada*

Received 17 March 1975. Accepted 19 May 1975

**Summary.—Antisera to extracts of a variety of bronchogenic carcinoma were raised in rabbits and extensively absorbed with immunoadsorbents prepared with normal lung extracts cyanogen bromide linked to Sepharose 4B, and glutaraldehyde insolubilized normal lung extracts. The antisera were tested by immunodiffusion against a panel of extracts from a variety of bronchogenic carcinoma, foetal lung extracts and pools of normal lung extracts. The results indicate that two distinct antigenic components are associated with bronchogenic carcinoma; one which is present in a high percentage of the tumour extracts tested and appears to have partial identity with a foetal lung component, and one (or more) which is not foetal and appears to have higher cross-reactivity (but not exclusively) with tumours of the same pathological type. Attempts to detect either antibody or antigens relating to these components in the serum of patients with bronchogenic carcinoma by these techniques were unsuccessful. The foetal cross-reacting component was neither carcinoembryonic antigen nor $\alpha_1$-foetoprotein.**

THERE HAVE BEEN a number of reports indicating that extracts from a variety of lung carcinomata contain antigenic components not demonstrable in equivalent extracts of normal human lung. Yachi and his co-workers (Yachi *et al.*, 1968) found that an ammonium sulphate (50% saturated) cut of tumour extracts contained 2 distinct antigenic components, one of which cross-reacted with a foetal antigen and one which appeared to be specific for tumour extracts. Neither antigenic component was demonstrable in all extracts tested. More recently, Mohr *et al.* (1974), using supernatants from tissue cultures of alveolar cell carcinoma as their antigenic preparation and absorbed sheep antiserum, demonstrated a common tumour specific antigen by immunodiffusion with the heterologous antiserum and cell culture supernatants as well as with serum from patients with alveolar cell carcinoma and, in some instances, with Hodgkin's disease. Sega *et al.* (1974) have reported the presence of tumour specific antigenic material present in a pooled extract of a variety of lung carcinomata. Similar observations have been made by others (Okada and Ikeda, 1970).

Although, to date, reports on the demonstration of tumour associated antigens in human bronchogenic carcinoma are not extensive, the indication in all the evidence so far published is that some common antigens may be present in this rather heterogeneous group of neoplasms. The present study was undertaken in an attempt to extend the body of knowledge to a point where some general conclusions might be drawn. Heterologous absorbed rabbit antisera to individual tumour extracts of a variety of pathological types of tumours were tested against a panel of 50 different tumour extracts, foetal extracts and extracts of either pooled or individual normal human lung tissues. The findings are reported in this paper.

### MATERIALS AND METHODS

*Extraction methods.*—All tissues were treated in essentially the same way. Tumours were obtained from either post mortem specimens or from surgical pneumonectomies; one tumour (C-24) was obtained as a metastatic tumour in the liver. They were prepared by excising all apparently normal tissue before grinding and extraction of the tumour. Normal lung preparations were obtained as post mortem specimens from patients dying from non-malignant disease. Foetal lungs were removed from 12–18 week saline induced aborted foetuses. Tissues were teased to separate individual cells and passed through a tissue grinder. The tissue volume was measured and 6 times the volume of 3·5 mol/l KCl was added, bringing the final salt concentration to 3·0 mol/l. Extractions were carried out with gentle agitation at 4°C for 18 h, after which the cell debris was removed by centrifugation at 16,000 *g* for 90 min. Cell extracts were dialysed exhaustively with several changes of phosphate buffered saline (PBS, 0·15 mol/l, pH 7·2), after which they were again centrifuged at 16,000 *g* for 90 min. The extracts were sterilized by Millipore filtration and stored at −20 °C.

Protein concentrations of the extracts were estimated by the standard Lowry technique. Tumour extracts contained between 5·0 and 15·0 mg/ml. Many of the normal extracts were maintained at considerably higher protein concentrations (30·0 mg/ml) in order to minimize the possibility of regarding relatively small quantitative differences in cell components as tumour specific materials.

*Antisera.*—Randomly bred albino rabbits were immunized by 8 weekly injections of tumour extract. Dose levels were at 7·5 mg of protein in 1·0 ml of 50% complete Freund's adjuvant, administered in 5 locations—4 intramuscularly in each limb and one intraperitoneally. Animals were test bled 10 days after the last injection by the ear vein and their serum tested by immunodiffusion with the homologous extract. If the antiserum, after immunoadsorption, failed to detect tumour associated material, further immunizations were carried out. Final bleeding was carried out by cardiac puncture. Serum was stored at −20 °C.

*Immunoabsorption.*—An immunoadsorbent was prepared by binding a pool of 8 normal lung extracts to Sepharose 4B with cyanogen bromide. The technique has been described in detail previously (Watson, Smith and Levy, 1974). The only changes in the basic technique was that the "anti-normal" antibody were eluted from the columns with Sorensen's buffer (glycine-HCl, pH 2·6), and the columns were regenerated with starting buffer.

Before the eluted anti-tumour antibody was concentrated, it was further absorbed on a glutaraldehyde insolubilized preparation of normal lung extract. This immunoadsorbent was prepared according to the method of Avrameas and Ternynck (1969) with the following modification: Normal lung extracts to be treated were dialysed against 0·1 mol/l acetate buffer pH 5·0 at 4 °C overnight. Glutaraldehyde at a concentration of 25% was added slowly, with stirring, to a final concentration of 50 mg/100 mg protein. The mixture was stirred at room temperature for 4 h, after which it was allowed to react for 18 h at 4 °C. The mixture was diluted 1 : 5 with PBS and centrifuged at 2000 *g* for 15 min at 4 °C. The pellet was homogenized and washed again with PBS, after which it was again homogenized and washed with 0·1 mol/l glycine-HCl buffer at 2·8. The insolubilized material was washed a further 2 times with PBS, homogenized and stored in PBS at 4 °C.

Absorption of the anti-tumour antiserum was carried out using a 1 : 1 volume of antiserum and insoluble normal extract. Absorption was carried out at 4 °C for 18 h, after which the insoluble material was removed by centrifugation. The supernatant was concentrated by either ultrafiltration on an XM 100 A ultrafiltration membrane (Amicon) or by precipitation with 50% saturated $(NH_4)_2SO_4$. The glutaraldehyde insolubilized immunoadsorbents were not re-used.

*Immunodiffusion.*—Immunodiffusion was carried out on glass slides using 0·85% Ionagar and a template for 6 samples around a centre well. Antiserum and extracts were added as 50 μl samples and diffusions were developed at 4 °C for 4 days in a humidified chamber. In some instances, titrations of extracts were run in which sample sizes varied between 10 and 100 $\mu$l, with volumes made up with PBS. Slides were washed in PBS for 3 days, dried, stained with Amido black and destained in 5% acetic acid for 30 min. Each series of 6 contained one sample of homologous extract and at least one normal or foetal extract.

*Tests for carcinoembryonic antigen (CEA) and $\alpha_1$-foetoprotein.*—Assays for CEA in tumour extracts in antisera were carried out by the Division of Nuclear Medicine, Vancouver General Hospital, using the CEA-Roche Test Kit.

Absorbed, highly specific goat antiserum to $\alpha_1$-foetoprotein was donated by Dr S. O. Freedman, McGill University School of Medicine. Tests with this antiserum were carried out by immunodiffusion using both amniotic fluid and foetal lung extracts as positive controls.

### RESULTS

A total of 15 absorbed heteroantisera were prepared in rabbits against extracts of individual tumour extracts of a variety of pathologies. These antisera were tested individually against a panel of 45 tumour extracts, 4 different normal lung extracts, one consisting of a pool of 8 normal lung extracts (referred to as normal pool), and 3 consisting of individual extracts and 2 foetal lung extracts each consisting of a pool of 8 foetal lungs. The immunodiffusion results are presented in Tables I–VI, and are broken down according to tumour pathology. The designation (i) used in these Tables indicates the presence of an immunodiffusion band on the inner region near the antiserum well. It was distinct from other outer immunoprecipitin lines (see below). Normal lung extracts, foetal lung extracts and normal serum samples were tested as controls. The results are presented in Table V. The percentage of cross-reactivity in relation to the pathology and the type of antiserum are shown in Table VI. All positive reactions recorded indicate positive immunodiffusion bands not seen in any of the normal extracts tested.

It can be seen that there appears to be some correlation between tumour pathology and the degree of cross-reactivity as demonstrated by this technique. A high degree of cross-reactivity was also seen between squamous cell carcinoma and anaplastic tumours. Some antisera were considerably more potent in the production of antibodies which were apparently tumour specific. It is not

TABLE I.—*Results of Immunodiffusion with Absorbed Antisera to Tumour Extracts of Various Pathological Types with Individual Extracts from Squamous Cell Carcinomata*

| Antigen extract (Squamous cell carcinoma) | Squamous cell carcinoma | | | | | | Adeno-carcinoma | | Anaplastic carcinoma | | | | Oat cell carcinoma | | Alveolar carcinoma |
|---|---|---|---|---|---|---|---|---|---|---|---|---|---|---|---|
| | C-6 | C-53 | C-57 | C-63 | C-66 | C-71 | C-24 | C-26 | C-41 | C-46 | C-62 | C-67 | C-40 | C-78 | C-30 |
| C-1 | + | − | − | + (i) | − | − | − | − | − | − | − | − | − | − | − |
| C-6 | + | − | − | + | − | − | − | − | + (i) | − | + | − | − | + | + |
| C-53 | + | + | + (i) | + (i) | − | − | − | − | − | − | | + (i) | − | − | + |
| C-60 | − | + | + (i) | | − | − | − | − | + (i) | − | + (i) | − | − | − | − |
| C-61 | | + | + (i) | | − | − | − | − | + (i) | − | + (i) | − | − | − | − |
| C-63 | − | + | + (i) | + (i) | − | − | | | + (i) | − | − | + (i) | − | − | + |
| C-66 | + | − | + (i) | + (i) | − | − | | − | + (i) | − | + | − | − | − | |
| C-69 | − | − + | + (i) | + (i) | − | − | | − | + (i) | − | + (i) | − | − | + | − |
| C-71 | − | − | + (i) | + (i) | − | − | | − | − | − | + (i) | − | − | − | − |
| C-74 | + | + | + (i) | − | − | − | | − | + (i) | − | − | | + | − | − |
| C-75 | − | + | + (i) | − | − | − | | + | + (i) | − | − | | − | − | − |
| C-76 | + | + | + (i) | − | − | − | | − | + (i) | − | − | | + | + | − |
| C-79 | | − | + (i) | + (i) | − | + | | − | + (i) | − | + | − | − | + | − |
| C-87 | | + | + (i) | − | − | + | | − | + (i) | − | − | − | − | + | − |
| C-90 | | + | + (i) | − | − | − | | − | + (i) | − | + | − | − | − | − |
| C-93 | | + | + (i) | − | − | − | | − | + (i) | − | − | | | − | − |
| C-94 | | + | + (i) | − | − | − | | − | + (i) | − | + | | | + | − |

Table II.—*Immunodiffusion Results using Absorbed Antisera to Tumour Extracts of Various Pathological Types with Individual Extracts from Adenocarcinomata*

| Antigen extract (Adeno-carcinoma) | Absorbed antisera: Squamous cell carcinoma C-6 | C-53 | C-57 | C-63 | C-66 | C-71 | Adeno-carcinoma C-24 | C-26 | Anaplastic carcinoma C-41 | C-46 | C-62 | C-67 | Oat cell carcinoma C-40 | C-78 | Alveolar carcinoma C-30 |
|---|---|---|---|---|---|---|---|---|---|---|---|---|---|---|---|
| C-3 | − | − | − | − | − | − | − | − | − | − | − | − | − | − | − |
| C-5 | − | − | − | − | − | − | − | − | − | − | − | − | − | − | − |
| C-24 | − | − | − | − | − | − | + | − | − | − | − | + (i) | − | − | + |
| C-26 | − | − | − | − | − | − | − | + | − | − | − | + | − | − | + |
| C-36 | − | − | − | + (i) | − | − | + | + | + (i) | − | − | + | − | − | + |
| C-45 | + | − | + (i) | − | − | − | − | − | − | − | − | + | − | − | − |
| C-58 | − | − | − | − | − | − | − | − | + (i) | − | − | − | − | − | − |
| C-85 | | − | + (i) | + (i) | − | − | | − | + (i) | − | − | + (i) | − | − | − |
| C-88 | | − | + (i) | − | − | − | | − | + (i) | − | + (i) | − | − | − | − |

Table III.—*Immunodiffusion Results using Absorbed Antisera to Tumour Extracts of Various Pathological Types with Individual Extracts from Anaplastic and Oat Cell Carcinomata*

| Antigen extract | Absorbed antisera: Squamous cell carcinoma C-6 | C-53 | C-57 | C-63 | C-66 | C-71 | Adeno-carcinoma C-24 | C-26 | Anaplastic carcinoma C-41 | C-46 | C-62 | C-67 | Oat cell carcinoma C-40 | C-78 | Alveolar carcinoma C-30 |
|---|---|---|---|---|---|---|---|---|---|---|---|---|---|---|---|
| Anaplastic carcinoma | | | | | | | | | | | | | | | |
| C-41 | + | − | + (i) | + | − | − | + | − | + | − | + + (i) | + (i) | − | − | − |
| C-46 | + | − | + (i) | − | − | − | − | − | − | − | + (i) | + | − | − | − |
| C-56 | + | + | + (i) | + | − | − | − | − | − | − | + (i) | + (i) | − | − | − |
| C-62 | + | + | + (i) | | − | − | | | + (i) | − | + + (i) | − | − | − | − |
| C-67 | − | − | + (i) | + | − | − | | − | + | − | − | + (i) | − | − | − |
| C-92 | − | − | + (i) | − | − | − | | − | − | − | − | − | | − | − |
| Oat cell carcinoma | | | | | | | | | | | | | | | |
| C-40 | − | − | − | − | − | − | − | − | − | − | + (i) | − | + | − | − |
| C-64 | − | + | − | | − | − | | − | + (i) | − | − | − | − | − | + |
| C-65 | − | − | + (i) | + (i) | − | − | | − | + (i) | − | + | − | − | − | − |
| C-70 | − | − | + (i) | + (i) | − | − | | − | + (i) | − | + (i) | − | + | − | + |
| C-72 | + | + | + (i) | + (i) | − | − | | − | − | − | + (i) | − | − | − | − |
| C-78 | | + | + (i) | − | − | − | | − | − | − | − | | + | + (i) | − |

Table IV.—*Immunodiffusion Results using Absorbed Antisera to Tumour Extracts of Various Pathological Types with Individual Extracts from Tumours of Miscellaneous Pathology all Arising from the Lung*

| Extract | Absorbed antisera: Squamous cell carcinoma C-6 | C-53 | C-57 | C-63 | C-66 | C-71 | Adeno-carcinoma C-24 | C-26 | Anaplastic carcinoma C-41 | C-46 | C-62 | C-67 | Oat cell carcinoma C-40 | C-78 | Alveolar carcinoma C-30 |
|---|---|---|---|---|---|---|---|---|---|---|---|---|---|---|---|
| Alveolar carcinoma | | | | | | | | | | | | | | | |
| C-30 | − | − | + (i) | − | − | − | − | + | − | − | + | + | + | − | + |
| C-81 | | + | + (i) | − | − | − | | − | + (i) | − | − | − | − | − | − |
| Mixed squamous adenocarcinoma | | | | | | | | | | | | | | | |
| C-82 | | − | + (i) | + (i) | − | − | | − | + (i) | − | − | − | − | − | − |
| Carcinoid | | | | | | | | | | | | | | | |
| C-4 | − | − | + (i) | − | − | − | | − | − | | − | − | − | − | |
| Rhabdomyo-sarcoma | | | | | | | | | | | | | | | |
| C-77 | + | + | + (i) | − | − | − | | − | + (i) | − | + | | − | + | − |
| Metastatic squamous | | | | | | | | | | | | | | | |
| C-57 | + | − | + + (i) | + (i) | − | − | − | − | + (i) | − | − | + (i) | − | − | − |
| Mesothelioma | | | | | | | | | | | | | | | |
| C-54 | − | − | − | + (i) | − | − | − | − | + (i) | − | − | + (i) | − | − | − |

TABLE V.—*Immunodiffusion Results using Absorbed Antisera to Tumour Extracts of Various Pathological Types with both Individual and Pooled Extracts from Normal Adult Lung, Foetal Lung and Serum Samples from Normal Individuals*

| | Absorbed antisera | | | | | | | | | | | | | | |
|---|---|---|---|---|---|---|---|---|---|---|---|---|---|---|---|
| | Squamous cell carcinoma | | | | | | Adeno-carcinoma | | Anaplastic carcinoma | | | | Oat cell carcinoma | | Alveolar carcinoma |
| Extract | C-6 | C-53 | C-57 | C-63 | C-66 | C-71 | C-24 | C-26 | C-41 | C-46 | C-62 | C-67 | C-40 | C-78 | C-30 |
| N-25 | — | — | — | — | — | — | — | — | — | — | — | — | — | — | — |
| N-50 | — | — | — | — | — | — | — | — | — | — | — | — | — | — | — |
| N-83 | — | — | — | — | — | — | — | — | — | — | — | — | — | — | — |
| N-pool* | — | — | — | — | — | — | — | — | — | — | — | — | — | — | — |
| F-48† | — | — | — | + (i) | — | — | — | — | + (i) | — | — | — | — | — | — |
| Serum 1‡ | — | — | — | — | — | — | — | — | — | — | — | — | — | — | — |
| Serum 2 | — | — | — | — | — | — | — | — | — | — | — | — | — | — | — |

* A pooled preparation of 8 individual normal lung specimens.
† A pooled preparation of 8 individual foetal lung specimens.
‡ Sera were taken from normal individuals and tested for the presence of antigen.

TABLE VI.—*A Summary of Data Presented in Tables I-IV showing the Percentage of Positive Results between Pathological Groupings of Antisera and Tumour Extracts*

| Extracts | Anti-squamous (6) | | Anti-adeno-carcinoma (2) | | Anti-anaplastic carcinoma (4) | | Anti-oat cell carcinoma (2) | | Anti-alveolar carcinoma (1) | |
|---|---|---|---|---|---|---|---|---|---|---|
| (number) | Total | + (i) | Total | + (i) | Total | + (i) | Total | + (i) | Total | + (i) |
| Squamous (17) | 46·8 | 23·4 | 4·9 | 0 | 41·9 | 33·9 | 28·1 | 0 | 18·7 | 0 |
| Adeno-carcinoma (9) | 11·5 | 9·6 | 25·0 | 0 | 27·7 | 19·5 | 0 | 0 | 33·0 | 0 |
| Anaplastic (6) | 42·8 | 17·1 | 12·5 | 0 | 45·8 | 33·3 | 0 | 0 | 0 | |
| Oat cell (6) | 32·3 | 20·6 | 0 | 0 | 30·4 | 26·1 | 33·3 | 12·5 | 33·3 | 0 |
| Alveolar (2) | 27·2 | 18·2 | 33·3 | 0 | 37·5 | 12·5 | 25·0 | 0 | 50·0 | 0 |

The data are further broken down to indicate what percentage of positive cross-reactions can be attributed to the inner immunodiffusion band which shows partial identity with foetal lung extract F-48.

clear whether this is applicable to individual animal differences or to differences in tumour extracts.

It was clear that at least 2 distinct types of tumour associated material were detectable by these procedures. One type formed a distinct clear band close to the antigen well, and cross-reactivity with other extracts did not occur at a high frequency. Typical examples are shown in Fig. 1. The second type, when present, formed a band relatively close to the antiserum well and cross-reactivity with other tumour extracts was at a high frequency (see antisera for C-57 and C-41). This band is designated in the Tables as + (i). While the outer bands at no time showed identity with either normal or foetal extracts, the inner band in some instances showed partial identity with one foetal extract (F-48). This is illustrated in Fig. 2 and 3. Some extracts demonstrated the presence of both inner and outer bands, indicating that 2 separate antigens were responsible (Fig. 4).

The possibility was considered that the inner band could be identified as carcinoembryonic antigen (CEA). Tests run on extracts which were either positive or negative for the presence of this reactive material showed that no correlation existed between CEA levels and the presence of the inner precipitin band. A large selection of tumour extracts as well as normal lung extracts were tested for the presence of CEA by the standard CEA-Roche Test Kit. Many of the tumour extracts contained appreciable levels of

FIG. 1.—Immunodiffusion results using absorbed antiserum to C-62 against 6 extracts designated as follows: 1, C-62; 2, C-77; 3, C-78; 4, normal pool; 5, C-93; 6, C-94. While strong precipitin lines developed against the extracts in wells 2 and 6, no bands formed against the homologous extract. The protein concentrations per well for each extract tested were as follows: C-62, 0·33 mg; C-77, 0·39 mg; C-78, 0·33 mg; normal pool, 1·45 mg; C-93, 0·41 mg; C-94, 0·31 mg.

FIG. 2.—Immunodiffusion results using absorbed antiserum to C-41 against 6 extracts designated as follows: 1, C-41; 2, C-65; 3, C-70; 4, F-48; 5, C-62; 6, C-85. The inner band seen shows partial identity with the foetal extract, F-48. The protein concentrations per well for each extract tested were as follows: C-41, 1·05 mg; C-65, 0·32 mg; C-70, 0·28 mg; F-48, 0·72 mg; C-62, 0·33 mg; C-85, 0·27 mg.

FIG. 3.—Immunodiffusion results using a poorly absorbed antiserum to C-41 against 6 extracts designated as follows: 1, C-57; 2, C-66; 3, C-67; 4, normal pool; 5, C-68; 6, C-69. The inner precipitin line seen in all the tumour extracts is not seen in the normal extract. The protein concentrations per well for each extract tested were as follows: C-57, 0·43 mg; C-66, 0·28 mg; C-67, 0·24 mg; normal pool, 1·45 mg; C-68, 0·43 mg; C-69, 0·58 mg.

FIG. 4.—Immunodiffusion results using absorbed antiserum to C-78 against 6 extracts designated as follows: 1, C-78; 2, C-77; 3, C-78; 4, normal pool; 5, C-93; 6, C-94. Note the occurrence of both an inner and outer precipitin line in well 2. Protein concentrations per well for each extract tested were as follows: C-78, 0·33 mg; C-77, 0·39 mg; C-78, 0·16 mg; normal pool, 1·45 mg; C-93, 0·41 mg; C-94, 0·31 mg.

FIG. 5.—Immunodiffusion results using anti-$\alpha_1$-foetoprotein at a 1 : 3 dilution against 6 extracts designated as follows: 1, C-53; 2, normal pool; 3, F-48; 4, C-40; 5, C-3; 6, C-57. The protein concentrations per well for each extract tested were as follows: C-53, 1·10 mg; normal pool 1·45 mg; F-48 (foetal extract), 0·72 mg; C-40, 1·90 mg; C-3, 0·63 mg; C-57, 0·86 mg.

CEA (>100 ng/ml) whereas others contained very low levels. Because the assayed levels of CEA did not correlate in any way with those extracts showing the presence of the inner band, it was felt that it was unlikely that the antigen in this instance was CEA. Similarly, radioimmunoassays run on antisera showing the presence of antibody specific for this inner band indicated that no detectable levels of anti-CEA were present. This combined evidence led us to conclude that this component could not be CEA.

The possibility that this inner band was related to $\alpha_1$-foetoprotein was also investigated. Absorbed goat anti-$\alpha_1$-foetoprotein was run in immunodiffusion with foetal extracts, tumour extracts and normal lung extracts. Strong positive precipitin lines developed between the foetal extracts and the antiserum but no detectable reaction was observed with either normal lung or tumour extracts. A representative set of results is shown in Fig. 5. Tumour extracts were chosen which either showed the presence of the inner precipitin line (C-53, C-40 and C-57) or did not (C-3). In all instances no immunological reactivity was seen with any tumour extract with undiluted goat anti-$\alpha_1$-foetoprotein, while this antiserum at dilutions of 1 : 10 reacted strongly with foetal extracts.

The serum from patients with either metastatic bronchogenic carcinoma or patients following surgery for bronchogenic carcinoma was tested by immunodiffusion with either tumour extracts (homologous or heterologous) or the absorbed antiserum, in an attempt to detect either circulating antigen or specific anti-tumour antibody. No antigen or antibody was detectable in any instance. Normal sera tested in the same way were also negative (Table V).

### DISCUSSION

It is extremely difficult to assess critically the data presented here. If the most naïve interpretation is to be used, it would appear that bronchogenic carcinoma cells may contain at least 2 components which are antigenic in a xenogeneic species and which are either absent or present in very low concentrations in normal lung tissue. One of the components appears to be cross-reactive but not identical to a component present in the extracts of foetal lung. This component is neither CEA nor $\alpha$-$_1$foetoportein. When antibodies to this component were present in appreciable amounts in absorbed antisera, the level of cross-reactivity between tumour extracts was found to be very extensive (see antisera C-41, C-57 and C-62 in particular). The reaction of this antibody with foetal extracts, on the other hand, was very weak and not detectable in most instances, even though the protein concentrations in the foetal extracts were generally higher than in the tumour extracts. This would imply that this constituent is not present in high concentrations in foetal lung. The nature of this component requires considerably more investigation before further conclusions can be drawn. It is interesting to note that earlier investigators (Yachi *et al.*, 1968) made the observation that a high percentage of the bronchogenic carcinoma extracts that they were studying contained a component which shared partial identity with a foetal antigen.

The second antigenic component(s) observed in this study is impossible to evaluate at this time. Whether or not the degree of cross-reactivity observed with antisera and the tumour extract panel is reliable cannot be assessed. It is still not clear whether all the outer precipitin bands observed are identical antigens even for an individual antiserum. It is possible that a greater degree of cross-reactivity may exist than was observed here since the methods used here for antigen detection are not very sensitive. This is apparent from our not infrequent observation that an antiserum would show no specific precipitation with the homologous antigen but would show significant precipitation with a heterologous tumour extract (see Fig. 1). The limit for detection of antigenic materials by the immunodiffusion technique used here is in the region of $2 \cdot 0$ $\mu$g/ml. It is possible that hyperimmunized rabbits could mount a considerable immune response to components in the extract present in concentrations lower than this level, and thus give rise to antisera which demonstrate tumour associated antigens in heterologous extracts (which contained higher concentrations of the antigen) but fail to do so with the homologous extract. It is also important to recognize the possibility that bacterial or viral contaminating antigens may be responsible for the formation of these bands since tumour specimens taken from the lung are invariably heavily contaminated.

The control tests included 3 individual extracts from lung tissue taken at post mortem from patients dying from non-malignant causes, as well as a pooled extract from 8 other individuals. In no instance did these extracts demonstrate positive immunodiffusion bands with the absorbed antisera which were reported as positive in this study. It is clearly impossible to ascertain at this point whether these data indicate qualitative or quantitative differences between lung tumour tissues and normal lung tissue.

Until these antigenic components have been purified and high titre monospecific antiserum prepared to them, these uncertainties cannot be clarified. Preliminary studies indicate that such an undertaking is possible and work is currently under way which addresses itself to these issues.

We would like to thank Dr Mincey and Ms B. L. Archibald, Division of Nuclear Medicine, Vancouver General Hospital (V.G.H.), for their cooperation in carrying out tests for the presence of CEA and for supplying materials for further CEA tests, and Dr S. O. Freedman for his gift of goat anti-$\alpha_1$-foetoprotein antiserum. We also thank Dr Peter Coy, British Columbia Cancer Institute, for obtaining both patients' sera, pathology reports and surgical tumour specimens, and Dr J. Burton, V.G.H., for obtaining post mortem specimens and autopsy reports.

### REFERENCES

AVRAMEAS, S. & TERNYNCK, T. (1969) The Cross-linking of Proteins with Glutaraldehyde and its Use for the Preparation of Immunoadsorbents. *Immunochemistry*, **6**, 53.

MOHR, J. A., NORDQUIST, R. E., RHOADES, E. R., COALSON, R. E. & COALSON, J. J. (1974) Alveolar Cell Carcinoma-like Antigen and Antibodies in Patients with Alveolar Cell Carcinoma and Other Cancers. *Cancer Res.*, **34**, 1904.

OKADA, Y. & IKEDA, S. (1970) Some Antigens Specific to Lung Cancer and Lung Tissue. *Kyoto Univ. Chest Dis. Inst. Bull.*, **3**, 113.

SEGA, E., NATALI, P. G., RICCI, C., MINEO, C. T. & CITRO, G. (1974) Lung Cancer Tumour Associated Antigen: Isolation by gel Filtration and Characterization by Immunodiffusion. I.R.C.S., **2**, 1278.

WATSON, R. D., SMITH, A. G. & LEVY, J. G. (1974) The Use of Immunoadsorbent Columns for the Isolation of Antibodies Specific for Antigens Associated with Human Bronchogenic Carcinoma. *Br. J. Cancer.*, **29**, 183.

YACHI, A., MATSUURA, Y., CARPENTER, C. M. & HYDE, L. (1968) Immunochemical Studies on Human Lung Cancer Antigens Soluble in 50% Saturated Ammonium Sulfate. *J. natn. Cancer Inst.*, **40**, 663.